# Motion Artifacts in Dynamic EEG Recordings: Experimental Observations, Electrical Modelling, and Design Considerations

**DOI:** 10.3390/s24196363

**Published:** 2024-09-30

**Authors:** Alessandra Giangrande, Alberto Botter, Harri Piitulainen, Giacinto Luigi Cerone

**Affiliations:** 1 Laboratory of Neuromuscular System and Rehabilitation Engineering, Department of Electronics and Telecommunications, Politecnico di Torino, 10129 Turin, Italy; alessandra.giangrande@polito.it (A.G.); alberto.botter@polito.it (A.B.); 2 Faculty of Sport and Health Sciences, University of Jyväskylä, 40014 Jyväskylä, Finland; harri.t.piitulainen@jyu.fi

**Keywords:** electroencephalography, biomedical instrumentation, motion artifacts, the brain, EEG electrodes, EEG cap design, electrode-amplifier system modelling

## Abstract

Despite the progress in the development of innovative EEG acquisition systems, their use in dynamic applications is still limited by motion artifacts compromising the interpretation of the collected signals. Therefore, extensive research on the genesis of motion artifacts in EEG recordings is still needed to optimize existing technologies, shedding light on possible solutions to overcome the current limitations. We identified three potential sources of motion artifacts occurring at three different levels of a traditional biopotential acquisition chain: the skin-electrode interface, the connecting cables between the detection and the acquisition systems, and the electrode-amplifier system. The identified sources of motion artifacts were modelled starting from experimental observations carried out on EEG signals. Consequently, we designed customized EEG electrode systems aiming at experimentally disentangling the possible causes of motion artifacts. Both analytical and experimental observations indicated two main residual sites responsible for motion artifacts: the connecting cables between the electrodes and the amplifier and the sudden changes in electrode-skin impedance due to electrode movements. We concluded that further advancements in EEG technology should focus on the transduction stage of the biopotentials amplification chain, such as the electrode technology and its interfacing with the acquisition system.

## 1. Introduction

Among the brain technologies, electroencephalography (EEG) is the most suitable for investigating the cortical sensorimotor integration processes during dynamic tasks thanks to its excellent spatiotemporal resolution, high portability, and relatively low costs [1]. Recent hardware developments allowed for the acquisition of biosignals through wireless, miniaturized, and portable devices, extending the range of signal acquisitions also outside lab environments [2,3,4,5,6]. The opportunities arising from the availability of these devices are, however, not fully exploited in practice due to the frequent presence of motion artifacts corrupting dynamic EEG signals. These artifacts are undesired signals with an amplitude of even two orders of magnitude greater than one of the signals of interest, thus strongly compromising the correct interpretation of cortical signals [7,8]. In the vast majority of the cases, motion artifacts are time-locked to the performed movements and greatly variable in terms of shape, repeatability, and spectral content, thus being hard or impossible to remove [9,10]. Indeed, motion artifacts can be observed both at low frequencies as baseline shifts and at high frequencies as spike-like variations [8]. Therefore, post-processing techniques are not always effective in removing these artifacts, considering the relatively low typical EEG frequency bandwidth (0.1 Hz–100 Hz) [11]. Whilst wavelet-based or blind source separation techniques are robust techniques excelling in removing physiological and repeatable EEG artifacts (e.g., eye blinks), their effectiveness in the context of motion artifact removal collapses. Indeed, it remains obscure to what extent they exclusively remove artifacts, entirely preserving the content of the physiological brain signals [9,12]. Over the past years, different solutions have been proposed to mitigate the recording of motion artifacts, including the use of active electrodes [13]. Although active electrodes were particularly effective in rejecting power line interference arising from the capacitive coupling between connecting cables and power line source, they have been proven comparable to passive electrodes in reducing motion artifacts during dynamic recordings [14]. Conversely, they contribute to increasing the encumbrance of the acquisition system, limiting its portability and usability in dynamic contexts. Other innovative solutions preventing the rising of motion artifacts concern the development of detection systems based on textiles, as they showed a reduced sensitivity to motion artifacts. However, their use is strictly limited to hairless cortical regions (i.e., frontal and temporal areas) and, therefore, not compatible with comprehensive studies on the role of the parietal sensorimotor cortices in movement control [15].

Despite these efforts, the genesis of motion artifacts in EEG recordings still remains a poorly understood topic. Therefore, given the increasing interest in dynamic EEG recordings in naturalistic, dynamic conditions [16,17,18], it is crucial to gain a deep understanding and to model the basic phenomena leading to the genesis of motion artifacts to optimize existing technologies and to develop new solutions for high-quality EEG detection.

Biopotential signal acquisition can be affected by the mutual interaction and superimposition of multiple factors occurring at different stages of the recordings (e.g., experimental setup preparation, detection, and acquisition technology) [19,20,21]. Although it is difficult to disentangle the sources of motion artifacts in the experimental practice, a model-based approach describing the basic phenomena underlying the generation of motion artifacts is hereby proposed. Specifically, in the following dissertation, we aim to provide further insights into the role of acquisition electronics, connecting cables, and electrode technology in EEG recordings, both from analytical and experimental perspectives. To achieve this, we (i) carried out observations on EEG signals during real experiments, (ii) identified and modelled the possible artifact sources, (iii) designed customized EEG electrode systems aimed at showing the influence of the detection system’s features in EEG dynamic recordings, and (iv) performed a case study aimed at giving further grounds to the previously modelled phenomena behind the genesis of EEG motion artifacts.

## 2. Observations

Potential sources of motion artifacts can arise at each of the three main stages constituting a traditional biopotential acquisition chain [22,23]: (i) the skin-electrode interface (i.e., transduction stage), (ii) the electrode-amplifier connecting cables, and (iii) the electrode-amplifier system (i.e., acquisition stage). Other possible sources of artifacts affecting the EEG signals (e.g., eye movements and environment-related artifacts) were out of the scope of the current dissertation as they are either easily handled or can be treated as a particular case of the described ones. Following this approach, we were able to investigate the main factors that can influence the outcome of biopotential signal recordings. Firstly, the relative movement between the electrode and the skin creates a consequent alteration of the ion distribution at the electrode-skin interface that would be read as an additive artifact signal with respect to those of interest [19]. Secondly, due to triboelectric phenomena [24], the friction and deformation of the cable insulator caused by the movements of the cables generate an additive input voltage potential that will be amplified together with the signal of interest [25]. Thirdly, in case of poor electrode-skin contact (e.g., due to a brisk, partial detachment of the electrodes), movements might also trigger a modulation of the residual input-referred Power Line Interference (PLI). In the next sections, we provide some examples taken from the abovementioned artifactual phenomena based on the observations of real recordings. These examples will then be used as starting points for the following electrical modelling.

### 2.1. Artifacts Arising from Phenomena at the Electrode-Skin Interface

**Figure 1:**
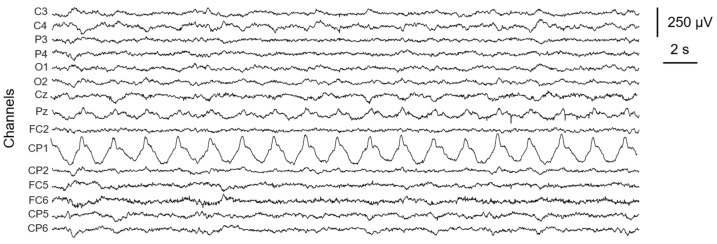
Examples of motion artifacts contamination on a set of EEG signals recorded during overground walking related to the movement of single exploring electrodes (CP1, Pz over the parietal cortex).

Figure 1 shows an example of motion artifacts corrupting individual channels (i.e., CP1 or, to a lesser extent, Pz of the parietal cortex) of a set of EEG signals recorded during overground walking. Such motion artifacts can be described as relatively slow changes in the baseline voltage potential highly correlated with the main frequency of the movement. In such cases, due to the slow and periodic changes of the voltage, we hypothesize that these artifacts are generated by relative shifts between the electrodes and the skin because of body movements related to the motor task. The artifact localization on a single channel is likely due to the movement of the individual exploring electrode (i.e., the electrode acquiring the monopolar EEG signal of interest with respect to the reference electrode). It is important to highlight that the example introduced in Figure 1 might be handled through post-processing techniques. However, if this type of artifact simultaneously affects multiple electrodes, including the reference one, the degree of signal corruption increases and the conventionally adopted techniques for artifact removal are critical to succeed due to the intricate superimposition of different effects.

### 2.2. Artifacts Related to Connecting Cables Movements

**Figure 2:**
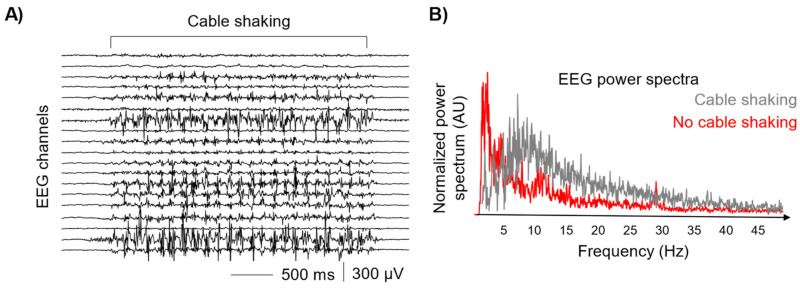
Motion artifacts caused by the movement of the connecting cables. (**A**) EEG signals recorded with the subject at rest while the experimenter is shaking the cables, wearing isolating insulating gloves. (**B**) Power spectra of a representative EEG signal with and without cable shaking.

Figure 2 represents the result of an experimental test regarding the acquisition of EEG signals from a subject at rest while the experimenter was manually shaking the cables connecting the electrodes to the amplifier (i.e., a worst-case scenario). As evident from the spectral power distributions of Figure 2B, traditional signal processing techniques cannot be used to dampen the dramatic effect of motion artifacts on EEG signals. Indeed, motion artifacts related to the connecting cables typically occur not time-locked with the movements with a spike-like behavior and their spectral components are overlapped with the EEG bandwidth (0.1 Hz–100 Hz). Additionally, motion artifacts generated by the movement of the cables are hardly repeatable, especially in terms of shape. For these reasons, many filtering techniques are not found to be effective in removing non-brain activity from the EEG signals [12]. Similar considerations have been observed in the case of sEMG signals acquisitions [21].

### 2.3. Artifacts Related to the Electrode-Amplifier System Properties Leading to PLI Modulation

**Figure 3:**
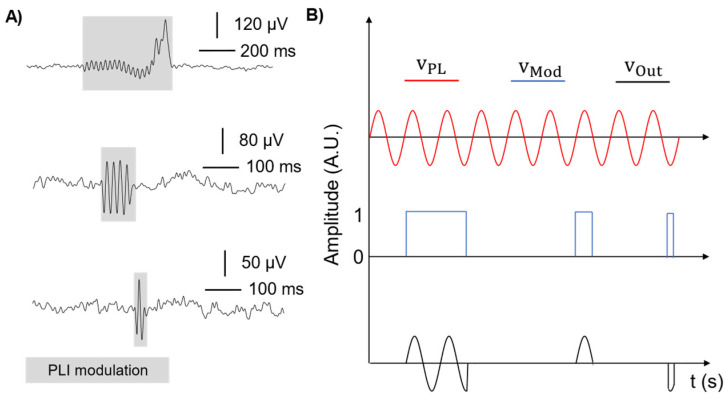
Examples of PLI modulation. (**A**) Three real examples of possible artifact morphology due to the brisk detachment of electrodes modulating power line noise. (**B**) Schematic representation of the hypothesized phenomenon. From top to bottom: power-line signal (red trace), modulating signal modelling the brisk electrode movement (blue trace), resulting detected signal (black trace) that will be superimposed to the physiological one.

Figure 3A shows experimental examples of artifacts due to PLI modulation on detected signals. In this case, we hypothesized that during movement, an unstable contact at the electrode-skin interface may induce a sudden variation of the electrode-skin imbalance between the exploring and reference electrodes, leading to a temporary increase of the input-referred PLI. Figure 3B represents a schematization of this phenomenon. The residual, input-referred PLI (red sinusoidal signal) is modulated by the movement (represented as the blue binary signal where the levels 0–1 are respectively referred to absence/presence of electrode-skin impedance variations due to a movement) providing in the output the corrupting signal (black color). Therefore, PLI signals (sinewave at 50 Hz/60 Hz) are modulated in time by the variations of electrode-skin imbalance, resulting in artifacts with different morphologies. As a result, the movement-related modulation is responsible for changing the spectral content of the whole recorded signal as it introduces spurious, unpredictable spectral components (different from PLI frequency) that may span throughout the entire EEG spectrum. These artifacts are, therefore, particularly challenging not only to be visually identified but also to be handled as they cannot be removed, e.g., through notch or adaptive filters [26].

## 3. Lumped Parameters Modelling

For each identified source of motion artifact (Observations 2.1, 2.2, and 2.3), an electrical lumped parameter model has been designed to describe and synthesize separately the experimentally observed phenomena.

### 3.1. Artifacts Arising from Phenomena at the Electrode-Skin Interface

**Figure 4:**
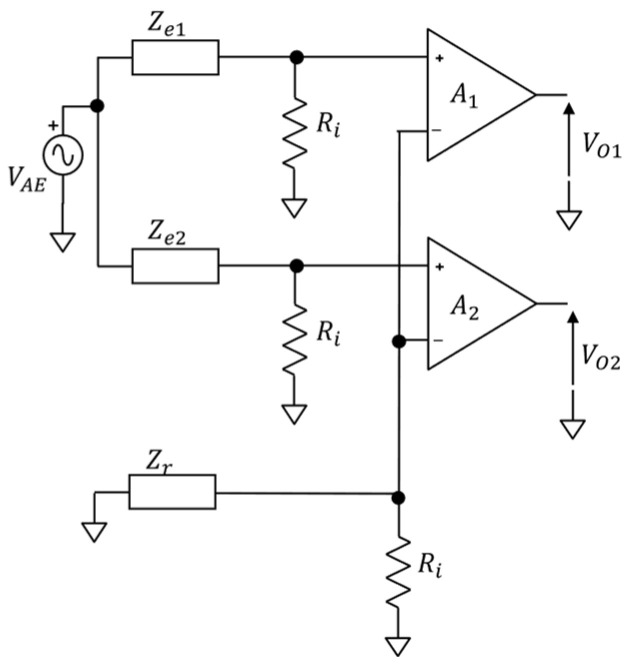
Electrical model of motion artifacts caused by movement-related shifts of two exploring electrodes ($e_{1}$ and $e_{2}$, with impedances $Z_{e_{1}}$ and $Z_{e_{2}}$). $R_{i}$ represents the amplifier input resistance. A differential signal acquisition in a monopolar configuration is represented.

Figure 4 shows the electrical model of generation of motion artifacts arising from the movement of two exploring electrodes $e_{1}$ and $e_{2}$ (having electrode-skin impedances respectively of $Z_{e1}$ and $Z_{e2}$) in the case of monopolar configuration (monopolar reference electrode $e_{r}$, having impedance $Z_{r}$). This circuit has been synthesized to model common artifacts between adjacent electrodes due, as an example, to movement-related shifts between the electrodes and the skin. A purely resistive amplifier input impedance is considered for simplicity [27,28,29]. The voltage generator ($V_{AE}$) models a common-mode motion artifact source as the voltage change generated by the relative movements between two exploring electrodes. This is assumed as a realistic hypothesis when considering, for example, two neighbouring electrodes affected by the same mechanical excitation. It is worth noting that in this electrical model, we considered a single pair of electrodes, but the dissertation can be extended to the total number of exploring electrodes used during EEG measurements. In addition, similar models can be used to examine the effect of the movements at the reference electrode location or both reference and exploring electrodes. We focused on this case because it is the most critical one in light of the abovementioned observations. According to the electrical model of Figure 4, the voltage divider between the front-end amplifier input impedances and the electrode impedances will generate the following input-referred voltages at the input of the A1 and A2 biopotential amplifiers:

(1) $$\left\{\begin{array}{c} V_{O1ir}=V_{AE}\frac{R_{i}}{R_{i}+Z_{e1}}\\ V_{O2ir}=V_{AE}\frac{R_{i}}{R_{i}+Z_{e2}} \end{array}\right.$$

To evaluate how the input common mode voltage artifact ($V_{AE}$) is translated into a differential-mode artifact at the amplifier output, we evaluated the difference between the two voltages $∆V=V_{O1ir}-V_{O2ir}$. Under the realistic hypothesis that the input amplifier impedance is greater than the electrode impedances (i.e., $R_{i}\gg Z_{e}$) [30], the voltage difference can be approximated as:

(2) $$∆V≅V_{AE}\frac{∆Z_{e}}{R_{i}}$$

This model is well known in the literature as the voltage divider effect, and it is often used to estimate the power line interference rejection capabilities of an electrode-amplifier system [21].

It is evident that, even when the source of the artifact ($V_{AE}$) is a common mode, the differential voltages computed at the output of the monopolar front-end may not be null, as they depend on the ratio between the electrode-skin imbalance and the amplifier input impedance. As a result, the difference between the impedance values $∆Z_{e}$ should be minimized as the greater the imbalance between the electrode impedances, the greater the voltage differences (i.e., artifact signal amplitude).

### 3.2. Artifacts Related to Connecting Cables Movements

**Figure 5:**
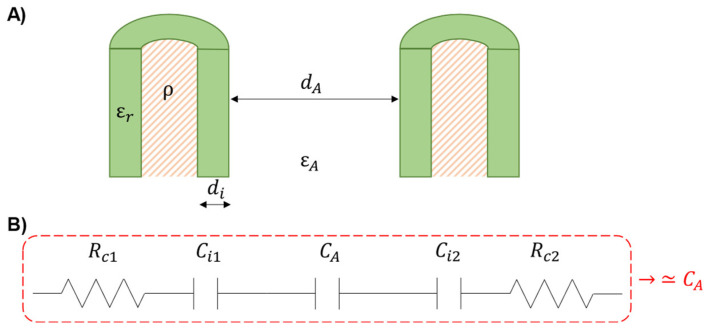
Model of two adjacent cables connecting EEG electrodes to the amplifier. (**A**) Schematic representation of the cross-section of two unipolar cables separated by a distance $d_{A}$ in a medium (air, dielectric constant $\varepsilon_{A}$). Each cable is composed of a conductive wire (resistivity ρ) embedded in an insulator sheath (thickness $d_{i}$, dielectric constant $\varepsilon_{r}$) (**B**) Equivalent electrical model of two adjacent cables, where $R_{c1,2}$ represent the electrical resistances of the conductive lead, $C_{i1,2}$ model the parasitic capacitances due to the cable insulator layer and $C_{A}$ depicts the electrical capacitance due to the dielectric $\varepsilon_{A}$. The red dashed rectangle indicates the simplified electrical model (≃$C_{A}$).

One of the major sources of cable-related motion artifacts is the triboelectric effect, causing a net charge accumulation on the surface of the cables connecting the electrodes to the amplifier during their reciprocal movements [25]. The triboelectric effect describes the transfer of electric charge between two objects (i.e., the insulation layers of neighbouring cables) when they slide against each other or even only when they come into contact [24,31]. Phenomena like friction and deformation of the insulation layers of adjacent cables modify the electrostatic voltage according to the cable material properties, contact area, type of contact, and speed of the varying reciprocal distance [25]. With the aim of understanding the contribution of the cables’ movement to motion artifacts in EEG recordings, we modeled two adjacent cables connecting two separated electrodes to the amplifier, as shown in Figure 5. $R_{c1},{\;R}_{c2}$ are the electrical resistances of the cable conductors (typically copper, resistivity $\rho ≅16.8\;m\Omega \cdot mm^{2}/m$), $C_{i1},C_{i2}$ model the parasitic capacitance due to the cable insulator layer (thickness $d_{i}$, dielectric constant $\varepsilon_{r}$) wrapping the inner conductive material, and $C_{A}$ represents the electrical capacitance due to the dielectric (i.e., air, dielectric constant $\varepsilon_{A}$) in between two conductive mediums (i.e., the charged insulator layers) separated by a distance $d_{A}$. Starting from this model, some simplifications can be conveniently introduced. First, the terms referring to the electrical resistances can be disregarded because of their small contribution to the impedance magnitude when considering standard cables with a transversal section of 0.5 mm^2^ and a length of 1 cm (~tens of milli-ohm). Second, under the hypothesis of modelling the reactive components as capacitors, the equivalent capacitance of the model can be approximated to the sole contribution of $C_{A}$ as it dominates on the single $C_{i}$ because of the greater dielectric constant ($\varepsilon_{r}>\varepsilon_{A}$) and distance between the plates ($d_{A}>d_{i}$). In addition, the movement of the cable bundle is expected to affect $C_{A}$ more than $C_{i}$. Indeed, the parameter $d_{A}$ (distance among cables) is most likely to vary throughout the movements, which modulates $C_{A}$. This, in turn, alters the total capacitance of the model, affecting the electrical properties of the cables and generating motion artifacts. Indeed, the triboelectric-induced electrostatic voltage consequently polarizes the $C_{A}$ capacitor, generating a voltage drop $V_{A}$. The net charge $Q_{A}$ on the plates of the capacitor (planar faces approximation) is proportional to the potential difference $V_{A}$ across the two plates:

(3) $$Q_{A}=C_{A}V_{A}$$

Under the reasonable assumption that the net charge, $Q_{A}$, accumulated through triboelectric effect remains constant during the cable movement, there will be a differential voltage change at the input of the biopotential amplifier $∆V_{A}=V_{A1}-V_{A2}$. Thus, the triboelectric-related voltage drop $∆V_{A}$ at the amplifier input can be modelled as an additive, purely differential mode voltage, added to the biopotential signal of interest. It is worth noting that this additive signal will be amplified by the differential gain which possibly leads to a relevant contamination to the recorded EEG signals.

### 3.3. Artifacts Related to the Electrode-Amplifier System Properties Leading to PLI Modulation

**Figure 6:**
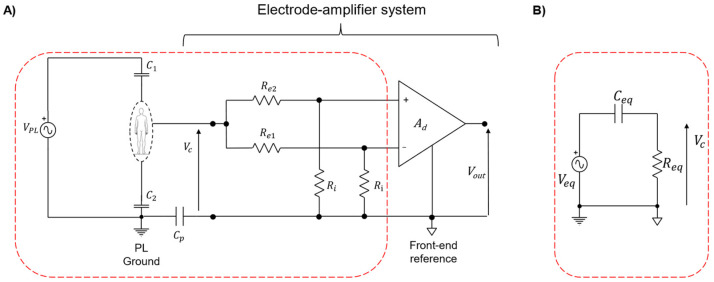
(**A**) Electrical model of the subject-electrode-amplifier system of a two-electrodes biopotential acquisition system. Ground-floating instrumentation and monopolar electrode configuration are represented together with a common mode excitation due to parasitic coupling between the subject and the power line ($C_{1}$ represents the parasitic capacitive coupling between the subject and the active phase of the power line, $C_{2}$ models the parasitic capacitive coupling between the subject and the power line ground). (**B**) Thevenin simplified the equivalent circuit of the power line-electrode-amplifier system when adopting battery-powered instrumentation.

Figure 6 shows the electrical model representing the parasitic coupling between a subject, the power line, and the electrode-amplifier system. It is used to model the PLI modulation phenomena as a source of movement artifacts in case of a residual amount of PLI at the input of the biopotential acquisition chain. Ground-floating instrumentation and monopolar electrode configuration are represented together with a common mode excitation due to parasitic coupling between the subject and the power line [32,33,34,35]. It is well known that the degree of PLI affecting biopotentials depends on the common-mode voltage at the input of the electrode-amplifier system. This voltage is mainly due to the parasitic capacitive coupling between the subject, the power line source and the ground, and to the coupling between the front-end reference and the power line ground. With reference to Figure 6A: $C_{1}$ (typically ranging from 5 pF to 20 pF [21]) represents the parasitic capacitive coupling between the subject and the active phase of the power line [30]; $C_{2}$ (~50 pF to 10 nF [21]) models the parasitic capacitive coupling between the subject and the power line ground [21,36]; $C_{p}$ represents the parasitic coupling between the front-end amplifier reference and the power line ground and it ranges between ten pF and hundreds of pico-Farads [28,30,36]. The model of Figure 6 also includes $V_{PL}$ modelling the common mode excitation (i.e., power line source), $R_{e1}$ and $R_{e2}$ representing the resistive components of the impedance models of the exploring electrodes and the input resistances of the front-end amplifier ($R_{i}$) [28,37]. Given these assumptions, the common mode voltage at the input of the electrodes-amplifier system $V_{C}$ can be computed through the Thevenin equivalent circuit extracted from the electrical model of the power line- electrode-amplifier system (Figure 6B). Where:

(4) $$\left\{\begin{array}{c} V_{eq}=V_{PL}\frac{C_{1}}{C_{1}+C_{2}}\\ C_{eq}=\frac{\left(C_{1}+C_{2}\right)C_{p}}{C_{1}+C_{2}+C_{p}}\\ R_{eq}=\left(R_{e2}+R_{i}\right)⨁\left(R_{e2}+R_{i}\right) \end{array}\right.$$

Additional simplifications can be conveniently introduced. Indeed, the input impedance of the front-end amplifier circuit $R_{i}$ is in the order of Mega-Ohms, at least three orders of magnitude greater than the electrode-skin impedance $R_{e}$ (tens of kΩ if 1 cm2 Ag/AgCl are used) [21]. Therefore, since $R_{i}\gg R_{e}$, the equivalent resistance of the Thevenin electrical circuit is given by $R_{eq}≅\frac{R_{i}}{2}$. Specifically, when considering a realistic EEG recording under a multichannel configuration (i.e., $R_{eN}$ with N ranging from 8 to 128 channels) and considering the common monopolar configuration having the inverting input shared between the channels, the total resistance at the monopolar reference input is obtained as the parallel of all the input resistances of each channel, i.e., $R_{eq}≅\frac{R_{i}}{N}$. Furthermore, in practice, it is generally possible to reduce the common-mode input voltage $V_{C}$ by minimizing the value of the parasitic coupling $C_{p}$ between the amplifier’s reference and the ground. This result may be obtained by designing battery powered, ground-floating, and miniaturized systems [22,38]. Therefore, the most common case within the present dissertation context leads to $C_{eq}≅C_{p}$ since $C_{p}$ dominates over the combination of $C_{1}+C_{2}$. Under these assumptions, the magnitude of the common mode voltage transfer function at the input of the electrodes-amplifier system $V_{c}$ results:

(5) $$|V_{C}|=V_{eq}\frac{\omega \frac{R_{i}}{N}C_{p}}{\sqrt{1+{\left(\omega \frac{R_{i}}{N}C_{p}\right)}^{2}}}$$

where *N* is the number of EEG channels. Given these considerations on the common mode input voltage $V_{C}$, it is well-known [21] that it is converted into a differential voltage ${V_{IRN}}_{50}$ according to (6):

(6) $${V_{IRN}}_{50}=V_{C}\left(\frac{R_{eN}-R_{e1}}{R_{i}}+\frac{1}{CMRR}\right)$$

where the term ($R_{eN}-R_{e1}$) is the interelectrode-skin impedances imbalance, Ri and CMRR are the amplifier’s input resistance and the common mode rejection ratio (CMRR) respectively. Equation (6) enables practical considerations regarding the phenomenon of the movement-related modulation of $V_{IRN_{50}}$ described in Section 2.3. Indeed, $V_{IRN_{50}}$ depends on:

- The common mode input voltage ($V_{C}$), which depends on both the design of the amplifier (i.e., $R_{i}$ in cases in which a third zero-volt reference electrode is not used, CP, etc.) and on the experimental setup adopted during the recordings (i.e., electrodes preparation, coupling between the subject and the power line, etc.). Thus, it can vary according to the movements performed during the recordings. However, a varying common mode voltage is unlikely the cause of movement artifacts as its variation would have an effect, although potentially different, on all the channels and could consequently be removed e.g., through a common average offline referencing.
- The common mode rejection ratio (CMRR) of the amplifier and the input amplifier resistance ($R_{i}$), are, in turn, dependent on the design of the front-end amplifier. As a result, no movement-dependent changes on the CMRR nor on $R_{i}$ are expected to occur and therefore it cannot be the cause hindering the variation of the $V_{IRN_{50}}$ when a constant $V_{C}$ is applied.
- The electrodes-skin resistances imbalance $\left(∆R_{e}\right)$. This parameter is the only one that can explain the observed modulation of power line interference on specific channels. Indeed, at a single channel level, the electrodes-skin resistance imbalance is obtained from the relative difference between the resistance of the exploring electrode and the one taken as a reference for the monopolar signal detection $∆R_{e}=R_{eN}-R_{e1}$. When performing a movement, the single values of electrode impedances may be affected by the changes caused by alteration of the skin-electrode contact due to e.g., reciprocal movements between the electrode and the skin, thus strongly contributing to the conversion of the common mode excitation to a differential one.

## 4. Technological Developments

The previous sections on the sources of motion artifacts were aimed at analytically describing the main factors influencing the quality of EEG electrodes. We highlighted that two main aspects should be considered when dealing with the contamination of motion artifacts on EEG signals: the presence of connecting cables between the electrodes and the amplifier and the varying interelectrode impedances due to the electrode movements. The former aspect takes into account the artifacts generated by the movements of the cables (Section 2.2), while the latter includes those related to the phenomena occurring at the electrode level (i.e., Section 2.1 and Section 2.3). In the following sections, we will describe the development and application of two EEG caps specifically designed to experimentally investigate the sources of motion artifacts identified in the previous paragraphs. Two customized EEG electrode systems have been designed to isolate the sources of motion artifacts and test the hypothesis on artifact genesis. The first EEG cap, hereafter referred to as ET Cap, is a textile-based system with electrical connections embedded into the fabric to minimize the effects of cable movement. The second EEG cap, named Lobster Cap, consists of a flexible PCB-based EEG electrode system aimed at reducing the effect of both connecting cables and electrode movement.

### 4.1. ET Cap: Textile-Based EEG Electrodes System

**Figure 7:**
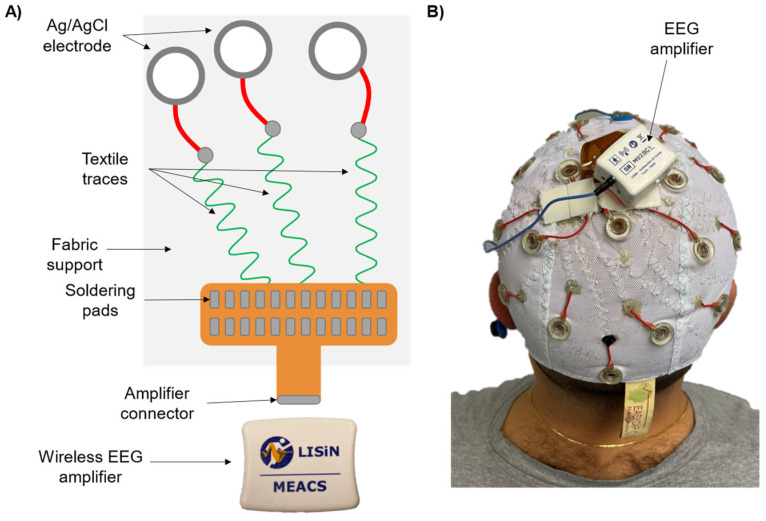
ET Cap. (**A**) Schematic representation of the textile traces sewed onto the EEG cap fabric and further connected to the exploring electrodes and the flexible connector constituting the input of the EEG amplifier. (**B**) Picture of a subject wearing the textile-based electrode system connected to the EEG amplifier.

Figure 7 shows the textile-based EEG electrode system (ET Cap). Figure 7A depicts the construction details of the textile traces. A commercially available EEG cap with thirty head-mounted electrodes (EasyCap GmbH, Gliching, Germany) was modified and adapted to our purposes. Specifically, the wires connecting the electrodes to the input amplifier have been replaced by conductive traces embedded in the fabric of the cap through a sewing machine. These traces are made of a conductive material coated with a thin insulating glaze (FIW diameter Ø = 0.1 mm, ELEKTRISOLA, Reichshof, Germany). The traces were incorporated into the textile substrate using a sinusoidal design to enhance the stretchability and reduce the mechanical stress on the wires during movements. Sinusoidal-shaped traces have been connected between a residual part of the electrode native cable (~5 mm), and the soldering pads of a flexible printed-circuits adaptor housing an FFC connector used to connect the cap with the MEACS EEG acquisition system [4,39] (ReC Bioengineering Laboratories and LISiN, Turin, Italy). To strengthen the welding points and to prevent their disruption, the electrode-textile traces connections were further reinforced through an epoxy adhesive glue (Pattex Power Epoxy), while the soldering pads of the connector side were covered with a thin layer of silicone (RS PRO, Corby, UK). The flexible PCB adapter was fixed onto the cap itself to minimize the cable lengths with the aim of placing the acquisition system on top of the head (see Figure 7B for details). Since the electrode technology was not changed with respect to the native one, the site preparation followed the practices in force for a standard EEG cap [21,30,40].

### 4.2. Lobster Cap: Flexible PCB-Based EEG Electrodes System

**Figure 8:**
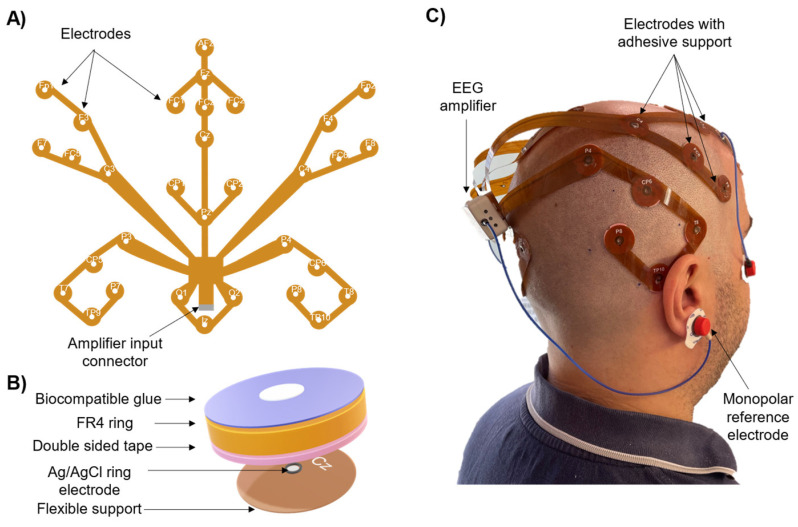
Lobster Cap. (**A**) Design of the entire system based on printed circuits on a flexible support. (**B**) Details of a single electrode preparation. An FR4 ring is attached on top of the electrode through a double-sided adhesive tape. Electrodes are then attached to the scalp by means of a biocompatible glue. (**C**) Picture of a subject wearing the Lobster Cap connected to the EEG amplifier.

Figure 8 depicts the flexible PCB-based EEG electrode system (named Lobster Cap due to its shape, Figure 8A). It is a two-dimensional flexible system of electrodes with both electrodes and traces integrated into a flexible polyimide substrate (80 μm thick). The flexible printed circuit connects thirty silver ring-shaped electrodes (inner diameter Ø = 0.8 mm) to the input connector of the EEG acquisition system. Each electrode site is labelled according to the 10–20 system as they are intended to be placed on the subject’s scalp according to the standardized positions identified and marked prior to the measurements. The Lobster Cap was designed to facilitate the adhesion of its branches to the subject’s scalp to prevent electrode movements, thus limiting cables and electrode movements, identified as causes of movement artifacts (Section 2 and Section 3). Figure 8B reports the detail of a single electrode showing three main layers allowing its adhesion to the scalp: a first double-sided adhesive tape (1 mm thick), an FR4 ring (~1 mm thick) needed to give mechanical support and to facilitate its bonding to the scalp through a final layer of biocompatible glue (Histoacryl, Braun Medical, Melsungen, Germany) used to further fix the electrodes in the correct position and avoid their relative movement with respect to the scalp. Finally, the electrode design included a ring shape allowing the user to inject the conductive gel after the electrode placement. Because of its design, the Lobster Cap is characterized by the total absence of free-to-move electrodes and connecting cables. It can also fit different head circumferences since the length of the different branches of the electrodes system are purposely designed to have a small slack allowing for a correct placement according to the subjects’ scalp size. Figure 8C shows the final stage with the Lobster Cap applied to a subject. It is important to underline that the designed solution is not intended to be used in a wide range of standard EEG measures due to the fact that it can be used only on bald subjects and the electrode preparation is complex and time-consuming. Instead, it was specifically designed as a research tool to study the generation of motion artifacts by mitigating two of their primary causes: movement of the cables and electrodes.

## 5. Case Study

An experimental study was carried out to demonstrate the hypotheses on the genesis of motion artifacts in dynamic EEG. Since it is not possible to experimentally separate the abovementioned three possible sources of motion artifacts, a mixed effect is expected to occur as an effect of cable- and electrode-related artifacts on EEG signals during movements. To this end, we detected EEG signals during dynamic motor tasks with both standard (i.e., wet electrodes with connecting cables to the amplifier) and the customized caps described in the previous section (i.e., ET Cap and Lobster Cap prototypes). Time- and frequency-domain variables were extracted to investigate the influence of cables and electrode technologies to give further grounds and experimentally investigate the hypotheses modelled in the previous sections.

### 5.1. Experimental Design

**Figure 9:**
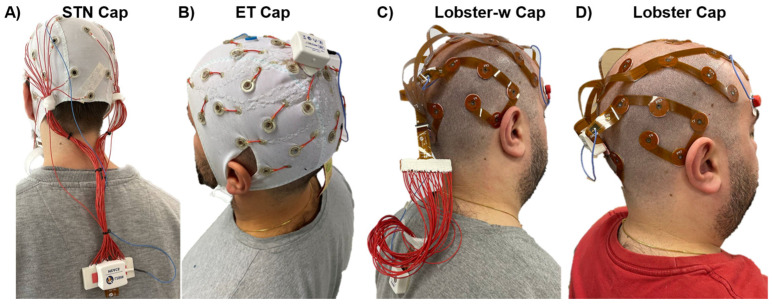
EEG electrodes systems used in the experimental case study: (**A**) STN—standard head-mounted electrodes with connecting cables, (**B**) ET Cap—textile-based system, (**C**) Lobster-w Cap flexible PCB-based system with connecting cables, (**D**) Lobster Cap flexible PCB-based system.

**Figure 10:**
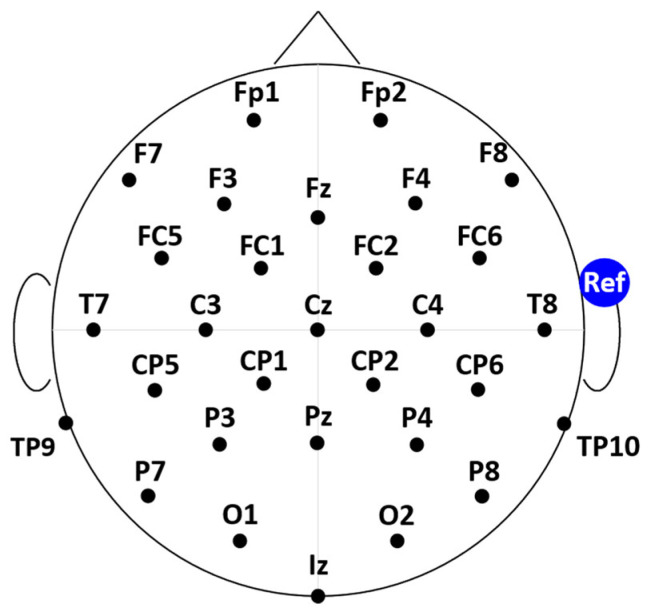
Schematic illustration of the electrode layout used for the study (30 EEG channels with the reference electrode placed on the right ear lobe). The table of coordinates can be found at [41]. Electrodes labels are reported according to the 10–20 system.

The study was conducted on a bald subject after having received approval from the University of Jyväskylä’s Ethics Committee in accordance with the Declaration of Helsinki. Figure 9 shows the four EEG electrode systems used in the study: (A) a standard cap (*STN Cap*), (B) the *ET Cap*, (C) the *Lobster-w Cap*, and (D) the *Lobster Cap*. The *STN* cap is a standard head-mounted electrode cap (EasyCap GmbH, Gliching, Germany) with 50 cm long cables between the electrode and the input connector of the acquisition system (Figure 9A); the *ET Cap* is the textile-based system (Figure 9B); the *Lobster-w Cap* is obtained from the Lobster Cap by adding a custom-made adapter constituted by 50 cm long connecting cables (Figure 9C); the *Lobster Cap* is the flexible PCB-based EEG electrodes system with no additional cables (Figure 9D). The four tested solutions were chosen to maximize the isolation of the identified sources of artifacts (i.e., cable movement and electrode shifts) in the experimental settings. Thirty EEG and two Electrooculograms (EOG) signals were recorded through a wireless EEG amplifier with a sampling frequency of 2048 Hz [2] (MEACS, ReC Bioengineering Laboratories and LISiN, Turin, Italy). The EEG channels involved in the recordings were the same for all four tested EEG electrode systems following the layout of the standard EEG cap (EasyCap–BC-TMS-32). Electrodes were placed according to the 10–20 EEG system as reported in Figure 10—Schematic illustration of the electrode layout used for the study (30 EEG channels with the reference electrode placed on the right ear lobe). The table of coordinates can be found in Figure 10 [41]. The experimental setup also included a general-purpose acquisition unit collecting magneto-inertial signals (100 Hz sampling frequency) placed on the head to track its acceleration, while a second unit (DuePro, OT Bioelettronica, Turin, Italy) was used to collect an additional analog signal from a footswitch (force sensor-FlexiForce A201, Tekscan, Norwood, MA, USA) placed under the right heel (2048 Hz sampling frequency). The synchronization unit introduced in [2] was adopted to synchronize all the abovementioned signals. The experimental protocol dealt with the repetition of three tasks: 60 s of (i) standing balance considered as a rest condition, (ii) treadmill walking at 4.6 km/h, and (iii) jogging at 6 km/h. Measurements were carried out on four different days (one EEG cap per day) to avoid any influence of consecutive scalp preparations on electrode-skin impedances. Subject preparation was performed following the recommended steps of electrode site abrasion and conductive gel injection [42]. At first, an abrasive paste (NuPrep, Weaver and Company, Aurora, CO, USA) was used to gently scrub the scalp by abrading the entire surface. Afterwards, the EEG electrode system was positioned and a conductive gel (NeurGel, SPES MEDICA, Genova, Italy) was inserted into the electrode cavities. Two additional electrodes (30 mm × 22 mm, Ambu s.r.l., Ballerup, Denmark) placed in the upper-left and lower-right corners of the subject’s eyes were used to record EOG signals. Finally, a further adhesive electrode (Ø = 24 mm, Kendall, Covidien-Medtronic, Minneapolis, MN, USA) was placed on the right ear lobe after a gentle skin abrasion and it served as the monopolar reference site for the EEG signals recordings. This reference electrode positioning was chosen both to standardize the reference electrode technology among the tested conditions and to minimize its movements. Indeed, being EEG signals recorded in a monopolar signal configuration, artifacts generated by the movements of the reference electrode could be confounding factors for the current artifact analysis.

### 5.2. Data Analysis

EEG signals were initially evaluated through visual inspection to exclude possible interference caused by poor electrode-skin contact to avoid this confounding factor. However, no missing contacts were found in the current data, and thus there were no rejected channels. In addition, we did not apply standard artifact correction algorithms, such as independent component analysis, to keep intact both the artifacts and the brain signal. We used the Matlab Software R2022b (Mathwork Inc., Natick, MA, USA) to carry out the analyses both in time and frequency domains that are described in the following. Statistical analyses were performed using R Statistical Software (v4.1.2; R Core Team 2021). EEG signals were acquired with a bandwidth between 0.1 Hz and 500 Hz [2]. They were further pre-processed offline through a fourth-order Butterworth bandpass filter (0.1 Hz–100 Hz), and then a common average reference approach was used to subtract the average of all the channels from each individual EEG channel. For the offline re-referencing, we did not include the initial reference when computing the average signal to correct for the intrinsic rank deficiency of the monopolar referenced EEG data [43]. However, although it does not ensure a full rank of the data, it does not affect our evaluation in this study.

**Figure 11:**
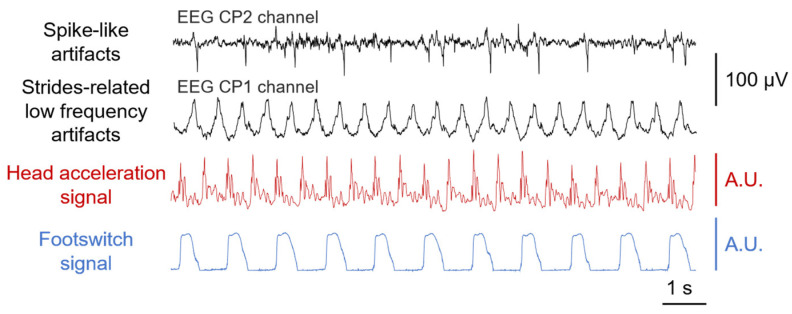
Examples of recorded EEG motion artifacts: spike-like and strides-related low-frequency artifacts recorded through a standard EEG electrode system. The head acceleration signal (Euclidean norm) and the force signal collected from the right heel are also reported timewise.

In light of what was discussed in the sections above, we expected signals corrupted by motion artifacts to be characterized by greater amplitudes. We therefore estimated the median Root-Mean-Square (RMS) value over 1-s epochs (50% overlap) for each signal. A one-way repeated measures ANOVA statistical test (Tukey’s post-hoc correction) was applied to the RMS values to evaluate the effect of the EEG electrodes system on the recorded signal amplitudes. Afterwards, we identified two types of motion artifacts corrupting EEG signals and they are shown in Figure 11 as an example taken from the experimental observations. The former artifacts are characterized by spurious spike-like motion artifacts most likely generated by the movements of the cables, whereas the latter artifacts are displayed as strides-related low-frequency variations and they are likely due to the movements of the electrodes (Section 2.1 and Section 2.2).

On one hand, to better investigate the influence of the cables, we compared the results of “cabled” electrode systems (STN and Lobster-w caps) to those of “non-cabled” caps (ET and Lobster). Therefore, the comparison was performed between the STN vs. ET caps and between the Lobster-w vs. Lobster Cap, having the same electrode technology. We hypothesized the presence of spurious spike-like artifacts to result in a heterogeneous distribution of amplitude values among EEG channels according to what was observed in Figure 2.

Thus, we first assessed the median kurtosis value of the amplitudes of the recorded EEG signals over 1 s long epochs (50% overlap). Then, the heterogeneity of the kurtosis values within the 30-EEG channels of the different electrode systems was quantified by calculating the variation coefficient for each kurtosis distribution. On the other hand, the effect of the electrode type was evaluated by comparing the results of the two custom-made solutions i.e., ET vs. Lobster caps. We expected repeatable artifacts time-locked to the heel strikes as shown in Figure 1 (i.e., strongly correlated with the gait frequency) when using standard electrodes (ET cap), with little to no strides-triggered artifacts when using electrodes attached to the scalp (Lobster cap). As a result, we used a two-step procedure to further assess the effect of the electrodes. At first, the wavelet coherence between cortical signals and the head acceleration signal was computed using the Matlab function coherence with default parameters (Morlet wavelet, 12 voices per octave, 15 octaves). The coherence spectra were averaged over time to identify the six EEG signals (i.e., 20% of the total amount of available channels) showing the highest coherence values. Afterwards, we extracted the average cortical response of the most coherent channels through a spike-triggered averaging technique with respect to the right heel strikes. Thus, the peak-to-peak amplitude values of the averaged response were finally compared to quantify the effect of electrode-related motion artifacts.

### 5.3. Results

**Figure 12:**
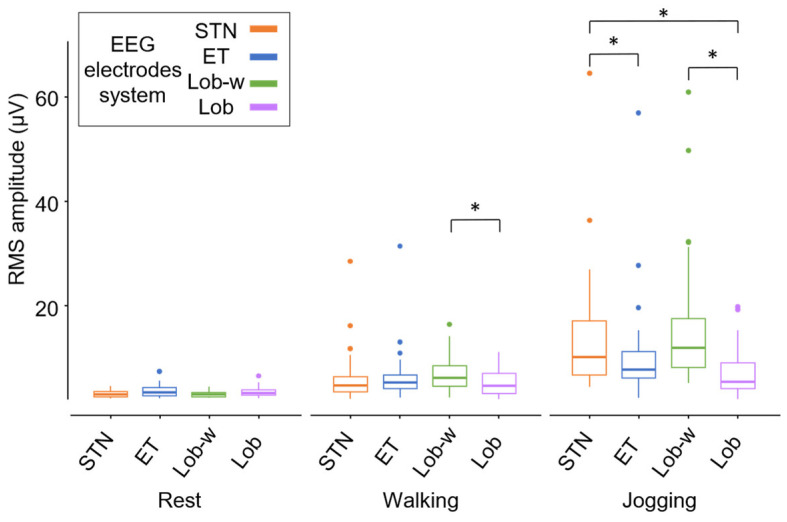
Boxplot of RMS amplitude values of 30 EEG signals recorded with the four electrode systems during rest, treadmill walking, and jogging. * *p* < 0.05 obtained with one-way ANOVA (Tukey post-hoc correction).

Figure 12 shows RMS amplitude values of the EEG signals recorded with the four electrode systems during rest, treadmill walking, and jogging. No statistically significant differences were found among RMS values of EEG signals recorded through the four electrode systems with the subject at rest or performing a treadmill walking task, except for the comparison between the cabled vs. non-cabled electrodes system (i.e., Lobster-w vs. Lobster). Results from the jogging task revealed, instead, statistically significant differences between RMS values obtained by cabled (STN and Lobster-w caps) versus non-cabled solutions (ET and Lobster caps). Additionally, further statistically significant differences were highlighted also between the STN versus the Lobster Cap.

**Figure 13:**
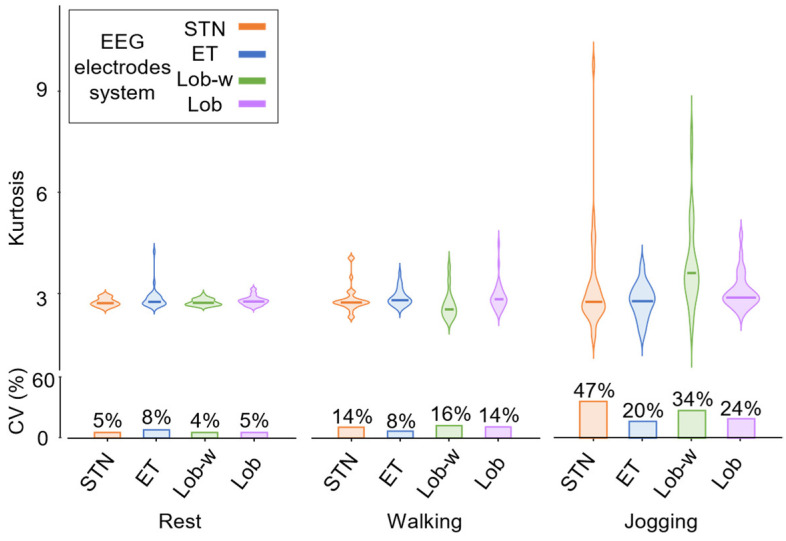
Top panel: violin plots displaying the median values of kurtosis computed over 1-s epochs for 30 EEG signals recorded through the four electrode systems in all the performed tasks. Bottom panel: bar diagrams of coefficients of variation (CV) of kurtosis values over the EEG electrodes in all the performed tasks.

Figure 13 represents the kurtosis values distributions and their coefficients of variation calculated on the 30 EEG signals with the four electrode systems in all the performed tasks. According to our hypotheses, the greater the task dynamics and the resulting cable movements, the more heterogeneous the amplitude signal distribution because of a higher occurrence of spike-like artifacts. Therefore, the highest RMS values accompanied by the greatest variation coefficients of the kurtosis values were expected for signals recorded through cabled electrode systems during the jogging task. In line with these expectations, although it is not possible to robustly disentangle cable and electrode effects in the experimental practice as they both contribute to an overall increase of the recorded signal amplitudes, these observations suggest that the most discriminating factor influencing the EEG signals amplitude is the movement of the cables. Indeed, Figure 12 and Figure 13 demonstrated that STN and Lobster-w caps (i.e., cabled) showed on average higher RMS amplitudes with a wider distribution according to the increase of task dynamics.

**Figure 14:**
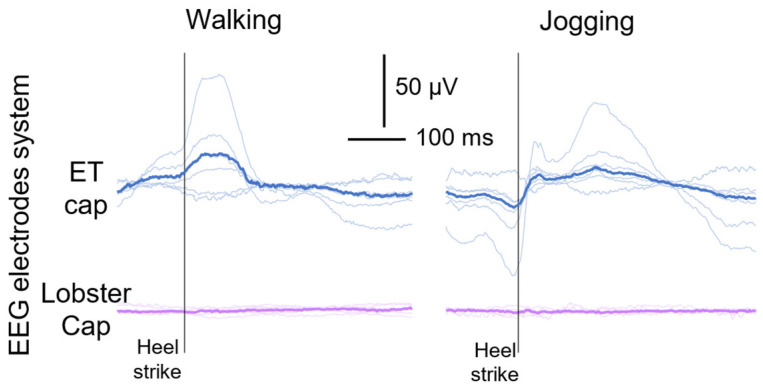
Averaged cortical responses with respect to the right heel strike onset were obtained from EEG signals recorded through the ET cap (blue traces) and the Lobster cap (violet traces) during walking and jogging motor tasks. Only the most 6 coherent EEG signals with the head acceleration are displayed.

Figure 14 shows the cortical responses averaged with respect to the right heel strikes across strides (n = 54 walking, n = 70 jogging) considering the most coherent channels with head acceleration during walking and jogging. Although different morphologies of cortical responses (e.g., even showing different polarities) might occur at the intra-electrode level, we found motion artifacts time-locked to the heel strikes, with high intra-electrode repeatability. In line with our expectations and discussions at the electrical modelling level, higher peak-to-peak amplitude values were obtained for the cortical responses recorded with the ET cap when compared to the Lobster cap (24.70 µV vs. 7.23 µV and 46.03 µV vs. 7.64 µV respectively during walking and jogging).

## 6. Discussion and Conclusions

The present study delved into the investigation of the genesis of motion artifacts collected during dynamic EEG recordings. An in-depth analysis of the underlying phenomena through electrical models and experimental tests has been performed. Given the availability of miniaturized and wireless EEG acquisition systems, the analytical approach highlighted two residual sites responsible for motion artifacts contamination of EEG signals: (i) the connecting cables between the electrodes and the amplifier and (ii) the sudden changes of electrode-skin impedance due to the electrodes movements. It is worth noting that the conducted experimental setup was not intended to separately investigate the analytically described sources of motion artifacts. Indeed, it is unlikely to experimentally disentangle the main causes of motion artifacts as a combined effect of cables and electrodes is expected to occur. Nevertheless, the experimental results showed that minimizing the length of the EEG electrode systems connecting cables and ensuring stable electrode contacts mitigates the EEG signal motion artifacts. Therefore, this outcome contributed to endorsing the analytical study of the phenomena hindering the genesis of EEG motion artifacts.

The observed case study was performed only on a single subject. Although this may be considered a possible limitation of the experimental part of this work, it is important to underline that the aim of the study is not to study the collection of movement artifacts among a population, but rather to validate a possible electrical modelling framework allowing to better understand possible sources of motion artifacts during EEG signals collection. Therefore, the primary aim of the study was to collect a set of EEG signals, without consideration of the physiological response underlying the studied tasks that would require a population of subjects.

Two customized EEG electrode systems have been designed and proposed. Data analysis on EEG recorded during dynamic tasks (i.e., walking and jogging) experimentally demonstrated that when the movements of both cables and electrodes are minimized, it is possible to record high-quality EEG signals even during dynamic movements. In light of what was obtained, practical considerations can be drawn up when dealing with EEG acquisition during movements:

- **Amplifier technology:** the state-of-art technology on miniaturized and wireless EEG acquisition systems seems to efficiently address the need for lightweight technology allowing for enough freedom of movement while recording brain signals [2,3,44]. In this regard, the use of active electrodes in the system electronics is intrinsically demonstrated not to provide an appreciable contribution in terms of mitigating motion artifact contamination on EEG signals. Indeed, their main contribution is to reduce the effect of capacitive coupling occurring downstream of the electrodes (e.g., parasitic capacitive coupling between connecting cables and power lines) [30]. On the contrary, their implementation becomes ineffectual towards electrode impedance imbalances occurring upstream the electrodes (i.e., $∆Z_{e}$ from (2)). This finding is in line with what was shown by Laszlo et al. [14] who experimentally showed that during rapid voltage fluctuations active electrodes are equally affected by movement artifacts related to changes at the electrode-skin interface with respect to passive electrodes. Conversely, the undesired result of using active electrodes in such contexts is the increase of the total system encumbrance and power consumption, thus contrasting with the need to develop miniaturized instrumentation.
- **Setup preparation:** Given that an ad-hoc preparation of the electrode sites is mandatory to ensure similar electrode-skin impedances magnitude among all the channels (i.e., to minimize $∆Z_{e}$ of (2) and (6)), it is also preferable to ensure a stable skin contact by avoiding temporary and brisk skin-electrodes detachments causing sudden electrodes impedance changes. This consideration applies also when dealing with the monopolar reference electrode as it affects all the recorded signals. Therefore, good practice recommendations regard the use of adhesive monopolar reference electrodes, preferably placed in body regions with limited movements (i.e., ear lobe). This is particularly important when recording electrophysiological signals under a monopolar signal configuration as perturbations additively interfering with the reference signal would affect all the channels. It could be hard to completely filter out these undesired perturbations e.g., by applying a common average filtering due to the superimposition of multiple confounding factors (i.e., additive noise, motion artifacts, etc.) simultaneously occurring at the level of exploring electrodes. In this regard, particular attention should be paid when applying re-referencing techniques, considering also possible processing-related needs [43].
- **Cap technology:** the choice of the EEG electrode system has a non-negligible influence on the quality of the collected signals in terms of motion artifact contamination. Indeed, as experimentally suggested by the proposed case study, the ideal case would be to keep the electrodes as fixed as possible such as in the case of the Lobster Cap. However, this type of solution, although optimal in terms of the quality of collected signals, holds intrinsic limitations from the applicability point of view: (i) it is usable only on either bald or short-haired subjects and (ii) it might require longer preparation times. However, considering the need for minimization of the connecting cable length and related reciprocal movements to mitigate the effects of triboelectric-related phenomena, embedding the connecting cables into the fabric of the cap of electrodes such as in the ET Cap could be a good compromise between usability and performance needs. Further technological advancements should therefore focus on the transduction stage of the biopotentials amplification chain such as the electrode technology and its interfacing to the acquisition system.

Although the present study focused on EEG signals during movements given their great clinical significance and relatively low signal-to-noise ratio, similar considerations may be applied to any biopotential acquired through surface electrodes.

In conclusion, the work presented herein constitutes a solid and widespread framework for modelling and understanding bio-electrical phenomena underlying the collection of motion artifacts during dynamic EEG. The insights, explanations and findings from this work could significantly contribute to driving technological developments and guide experimental setup practices in the field of dynamic EEG acquisitions.

## 7. Patent

An Italian patent application has been proposed by Politecnico di Torino for the ET Cap described in this study. All the authors have been recognized as inventors.

## Data Availability

The data are not publicly available due to privacy or ethical restrictions. However, data are available upon request from the corresponding author.
